# Nicotinic α4 Receptor-Mediated Cholinergic Influences on Food Intake and Activity Patterns in Hypothalamic Circuits

**DOI:** 10.1371/journal.pone.0133327

**Published:** 2015-08-06

**Authors:** Ana P. García, Teemu Aitta-aho, Laura Schaaf, Nicholas Heeley, Lena Heuschmid, Yunjing Bai, Francisco J. Barrantes, John Apergis-Schoute

**Affiliations:** 1 Department of Pharmacology, University of Cambridge; Cambridge, United Kingdom; 2 Laboratory of Molecular Neurobiology, Biomedical Research Institute (BIOMED) UCA–CONICET, Faculty of Medical Sciences, Catholic University of Argentina, Buenos Aires, Argentina; 3 Institute of Metabolic Science, University of Cambridge, Addenbrooke's Hospital, Cambridge, United Kingdom; 4 Key Laboratory of Mental Health, Institute of Psychology, Chinese Academy of Sciences, Beijing, China; CRCHUM-Montreal Diabetes Research Center, CANADA

## Abstract

Nicotinic acetylcholine receptors (nAChRs) play an important role in regulating appetite and have been shown to do so by influencing neural activity in the hypothalamus. To shed light on the hypothalamic circuits governing acetylcholine’s (ACh) regulation of appetite this study investigated the influence of hypothalamic nAChRs expressing the α4 subunit. We found that antagonizing the α4β2 nAChR locally in the lateral hypothalamus with di-hydro-ß-erythroidine (DHβE), an α4 nAChR antagonist with moderate affinity, caused an increase in food intake following free access to food after a 12 hour fast, compared to saline-infused animals. Immunocytochemical analysis revealed that orexin/hypocretin (HO), oxytocin, and tyrosine hydroxylase (TH)-containing neurons in the A13 and A12 of the hypothalamus expressed the nAChR α4 subunit in varying amounts (34%, 42%, 50%, and 51%, respectively) whereas melanin concentrating hormone (MCH) neurons did not, suggesting that DHβE-mediated increases in food intake may be due to a direct activation of specific hypothalamic circuits. Systemic DHβE (2 mg/kg) administration similarly increased food intake following a 12 hour fast. In these animals a subpopulation of orexin/hypocretin neurons showed elevated activity compared to control animals and MCH neuronal activity was overall lower as measured by expression of the immediate early gene marker for neuronal activity cFos. However, oxytocin neurons in the paraventricular hypothalamus and TH-containing neurons in the A13 and A12 did not show differential activity patterns. These results indicate that various neurochemically distinct hypothalamic populations are under the influence of α4β2 nAChRs and that cholinergic inputs to the lateral hypothalamus can affect satiety signals through activation of local α4β2 nAChR-mediated transmission.

## Introduction

The hypothalamus is a brain region important in sensing peripheral signals that relate to the energy requirements of an organism. Discrete neurochemically defined circuits respond differentially to peripheral cues to engage in or refrain from food-seeking behavior, depending on the body’s energy requirements [1–3]. For example, within the lateral hypothalamus (LH), genetic deletion of the orexin/hypocretin (HO) peptide results in a gain in weight in mice [4] whereas similar disruption in the melanin-concentrating hormone (MCH) leads to hypophagia and weight loss [5–7]. Neurons in the paraventricular hypothalamus (PVH), a subpopulation of which express oxytocin [8], have been shown to encode satiety states since their activation results in hypophagia. Likewise oxytocin infusions result in a significant decrease in food intake [9–10] together supporting a role for oxytocin in signaling satiety [11]. Hypothalamic dopamine transmission has similarly been shown to be important in energy balance [12–14] but the source of this dopaminergic regulation of hypothalamic function is currently unresolved. Within the hypothalamus two distinct dopaminergic populations reside in areas A13 and A12 in dorsal and ventral hypothalamic regions, respectively [15]. Their role in the regulation of energy homeostasis is unknown but their intrinsic hypothalamic projections [16] are suggestive of a functional influence on hypothalamic function.

Nicotine, an active ingredient in tobacco, is known to act as a potent anorectic, causing a decrease in food intake when administered both systemically and into discrete brain regions including the hypothalamus [17–20]. Using cell-specific receptor knockdown techniques, Mineur et al., [21] have demonstrated that this nicotine-mediated reduction in food intake is dependent on α3β4 nicotinic acetylcholine receptors (nAChRs) located on arcuate POMC neurons and their downstream melanocortin targets in the PVH. Systemic as well as local nicotine treatment, however, has been shown to activate a variety of neural circuits in the hypothalamus [21–25]. Moreover, ex and in vivo infusion of acetylcholine (ACh) has been shown to influence the electrical activity of neurons within and around the LH including HO, MCH, and neurons in the PVH [25–27]. Interestingly, Shimazu et al. [28], have shown that hypothalamic infusions of ACh can affect glycogen production by increasing liver glycogen synthase suggesting for a causal link between hypothalamic ACh transmission and energy conservation. Overall, these results indicate that ACh, the endogenous ligand for the nAChR, plays a role in regulating appetite and metabolism via its inputs onto various hypothalamic circuits.

The hypothalamus receives extensive cholinergic innervation from various sources including the substantia innominata [29], the laterodorsal and pontine tegmental nucleus (LDT, PPT, respectively) [30–31] and locally via hypothalamic choline acetyl transferase (ChAT)-containing interneurons [32–33]. In the brain, the predominant nAChRs are homomeric or heteromeric pentamers made up of the α7 or α4β2 subunits, respectively, the α3β4 nAChR being less common; all three types act functionally as cationic channels. Within the hypothalamus, the α4β2 is mostly expressed in the LH with some expression in the arcuate nucleus and PVH (Wada et al., 1989) whereas mRNA for the α7 subunit is expressed predominantly in the VMH but also in the arcuate, LH and PVH [34].

Studies that combined nicotine application with different nAChR antagonists have shown that nicotine can affect distinct hypothalamic circuits through activation of nAChRs with different subunit composition. Nicotine application in vitro has been shown to increase the inhibitory tone in hypothalamic neurons via the activation of both αβ [23] and α7 [27] nAChRs, as well as directly activate HO neurons [25]. Quantifying the transcription factor cFos, a molecular marker of activity, in HO neurons, Pasumarthi et al. [24] showed that systemic nicotine administration can increase HO activity. This nicotine-mediated activation of HO neurons is likely due to α4β2 nAChR-activation since in their study nicotine-mediated increases in HO cFos were shown to be disrupted by co-administration of di-hydro-ß-erythroidine (DHβE), an α4 receptor antagonist with moderate affinity, therefore implicating α4β2 nAChRs in the cholinergic regulation of hypothalamic function.

Despite much data supporting a role on nicotine-mediated suppression of appetite using exogenous nicotine adminstration the mechanism by which the endogenous nicotinic receptor agonist ACh in regulating appetite has received little attention. Due to the dense cholinergic innervation of hypothalamic regions with known influences on consummatory behavior we hypothesize that ACh acting on distinct hypothalamic circuits can regulate food intake. To characterize the mechanisms by which ACh neurotransmission within the hypothalamus regulates feeding behavior, animals were acutely fasted, systemically injected or infused with either DHβE or saline into the LH, re-introduced to chow and their food intake subsequently measured for two hours. Our results show that antagonizing hypothalamic α4β2 nAChRs result in animals consuming more food two hours post its reintroduction following a 12 hour fast. Immunocytochemical analysis showed that hypothalamic circuits known to be involved in regulating appetite express the nAChR α4 subunit, two of which, the HO and MCH systems, show differential activity patterns with intraperitoneal (i.p.) injections of DHβE compared to saline as indicated by cFos immunoreactivity. These results demonstrate that hypothalamic α4β2 nAChRs are engaged during consummatory behavior and may act to control food intake through actions on HO and MCH neurons.

## Methods

All procedures were carried out in accordance with the UK Animals (Scientific Procedures) Act 1986, and approved by the Local Ethical Review Panel at the University of Cambridge, UK (Project License 70/7548). 41 adult male Wistar rats (Harlan Laboratories Ltd.) weighing 300–400 g were individually housed in a standard bedded home cage with water and rat chow pellets available *ad libitum* in a light-controlled (12 h on/12 h off) and temperature-controlled (21.5–22.5°C) environment.

### Stereotactic surgery

Sixteen rats were anesthetized with isoflurane (induction 5%, 2% maintenance; Abbott Ltd, Maidenhead, UK), and bilaterally implanted with 22 gauge stainless steel guide cannulae (Plastics One, Roanoke, VA, US) into the LH using a stereotactic device (David Kopf Instruments, Tujunga, CA, US). Dental cement (Simplex Rapid, Associated Dental Products Ltd, Swindon, UK) and four stainless steel screws were used to secure the cannula placement. The coordinates used were 2.1 mm posterior to bregma, 2.0 mm lateral to midline, and 3.2 mm ventral to dura mater. Guide injectors were inserted to keep cannulae unobstructed during the recovery of 1 week. Animals’ recovery was monitored daily and none showed signs of distress. One animal was excluded from the dataset due to misdirected cannulae placements.

### Intracranial microinfusions

Bilateral infusion rate (0.25μl/min) was controlled by a syringe pump (kDScientific, Holliston, MA, US) that operated a microsyringe (Hamilton, Bonaduz, Switzerland). Polyethylene tubing connected the syringe to 28 gauge injectors (Plastics One) projecting 5 mm below the guide cannulae. DHβE or saline was infused over a 2 min period, and thereafter the cannulae were left in place for an additional 1 min to allow for diffusion of the saline or drug into the tissue before removing the injectors. The food intake study started immediately after the infusions.

### Behavioral testing

Two days prior to fasting, 20 animals were handled and given an i.p. injection (needle, 25 gauge) of saline (the volume of all injections was 1ml/kg unless otherwise stated) or in cannulated animals (n = 16) an infusion of sterile saline (0.50μl) daily for 2 consecutive days at the end of the dark phase [35]. Two groups of cannulated animals and three groups of i.p. injected animals were fasted for the 12 hour dark phase. When lights were turned on, each group of animals was given either an i.p. injection or intra-LH infusion of saline solution or different concentrations of DHβE (Tocris Bioscience) (for i.p. injections 1 and 2 mg/kg and for intra-LH infusions, 20 μg/side over 2 min) dissolved in saline solution. The DHβE doses used were based on effective doses from previous studies [35–37] Experimenter was blinded to the identity of the drug and animal conditions were counterbalanced for order. All injections were performed in a designated procedure room. After injections, a known amount of food was presented to each animal, and they were given free access to this food for 2–3 hours (Re-fed groups) in their home cages. After this period of time, the remaining food was weighed, and food intake over 1–2 hours calculated. Three hours post injection i.p injected animals were sacrificed and confirmed unresponsive by anesthetizing them with a lethal dose of sodium pentobarbital and checking reflexes with a paw-pinch, before intracardially perfusing them with PBS followed by 10% formalin. Brains were removed and kept in 10% formalin for 4 hours. All brains were sectioned (40 μm thick), and collected in 6 series in PBS treated with DEPC.

### Analysis of the expression of the α4 nAChR subunit in HO, MCH, TH and oxytocin neurons

The α4 nAChR and neuropeptide immunolabeling was done sequentially. A total of four animals were used for all α4 nAChR subunit immunolableing. In three animals, all four peptides were analysed. For the 4^th^ animal however we were unable to isolate a sufficient amount of A13, A12 tissue for TH immunolabeling. For the 4^th^ animal however we were unable to isolate a sufficient amount of A13, A12 tissue for TH immunolabeling. This was due to the overlapping distribution in the rostral-caudal axis of the different cell populations that we investigated. First, tissue for α4 nAChR immunolabeling was washed three time for 10 minutes in PBS (3 x 10 min), incubated in endogenous peroxidase by 10 min incubation in 0.9% H_2_O_2_ in methanol, 10 min wash in distilled water, 20 min incubation in 2% H_2_O_2_ in PBS, and a further wash in PBS. The tissue was then antigen blocked for 1 hour in PBS containing 3% BSA and incubated overnight with primary antibody (goat anti–α4 nAChR sc1772, Santa Cruz Biotechnology) at a concentration of 0.2μg/ml (1:500) in blocking buffer at 4°C. The next day, slices were washed and incubated with secondary antibody (biotinylated donkey anti–goat IgG, 1:200) in blocking buffer for 1 hour, re-washed and incubated with peroxidase–conjugated avidin–biotin complex (ABC) for 1 hour. Slices were washed before and after development in 0.04% 3.3’-diaminobenzidine (DAB, Vector Laboratories). Immediately after α4 immunolabeling the brain slices were washed 3 x 10 min in PBS and incubated in blocking solution (1% BSA and 0.3% Triton X–100 in PBS) for 1 hour. Slices were then incubated overnight with rabbit anti-orexin A antibody (1:1000: Phoenix Pharmaceuticals Inc), rabbit anti-MCH (1:1000: Phoenix Pharmaceuticals Inc.), mouse anti-TH (1:1500; Invitrogen), or rabbit anti-oxytocin (1:2000; Immunostar) at 4°C. The next day slices were washed and incubated with Alexa-Fluor 488 (donkey/goat) anti-rabbit or anti-mouse for 2 hours at room temperature, and then washed again thoroughly and finally mounted under coverslips in a Vectashield mounting medium for microscope observation.

### cFos-expression in neurochemically-distinct hypothalamic neurons

Tissue from experimental i.p injected animals was used for all cFos immunolabeling (saline, n = 6; 2 mg/kg DHβE, n = 6). For technical reasons we were unable to recover the brain of one control animal. The sample size differed between the different experimental conditions as a result of overlapping cell populations in the rostral-caudal axis that left very little room for methodological error and sufficient tissue for all cell populations to be analysed in all animals. Before proceeding with cFos staining, slices were washed before blocking for 1 hour in 0.3% H_2_O_2_ in PBS and incubated in blocking solution (3% BSA and 0.04% Triton–X-100 in PBS) for 1 hour at room temperature. The tissue was then incubated for 2 nights with a rabbit anti-cFos antibody (1:10,000) (Calbiochem) in blocking solution at 4°C. Following the incubation, slices were washed, incubated in biotinylated donkey anti–rabbit serum (Jackson Laboratories, 1:500) in blocking solution for 1 hour, and washed before being incubated with peroxidase—conjugated avidin—biotin complex (ABC) for 1 hour. Slices were washed before development in 0.04% 3.3’-diaminobenzidine (DAB; Sigma), following manufacturer’s instructions. Immediately after HO, MCH, TH, and oxytocin immunohistochemistry was carried out as described above and followed by a thorough washing in PBS before mounting with coverslips in a Vectashield mounting medium (Vector Laboratories).

### Microscopy

Slides were examined using a Zeiss Axioskop 2 light microscope. Bregma levels were determined for each slice, using a rat brain atlas for each brain slice to allow the identification of subregions of the hypothalamic area. Images were taken using a QImaging Fast 1394 Camera. Images were collected using a GFP filter to visualize fluorescent neurons, and DAB reactivity was visualized using bright-field microscopy.

### Analysis of images

The images of the brain slices from each animal were collated in folders, allocated a random number by an independent person and then analyzed using ImageJ (NIH) software. The counter was blinded as to which animal’s brain slice was being analyzed. For α4 nAChR subunit or cFos and HO, MCH, TH, oxytocin colocalization the α4 nAChR or cFos images were negatively inverted and false colored (red). The fluorescent and false-colored images were overlaid in Microsoft Powerpoint, and a brain outline scaled and superimposed onto the image to consistently identify the various hypothalamic nuclei and subdivisions. In ImageJ the “cell counter” plugin was used to count the total number of fluorescent neurons and those containing α4 nAChR or cFos. As there is no known lateralization of HO, MCH, oxytocin or hypothalamic TH function or activity, the images from both hemispheres were included in the analysis. Coronal slices between animals were matched as best as possible between animals and based our analyses on tissue from similar rostral-caudal levels. In all cases we calculated the percent of cell type that expressed cFos relative to total count of analyzed cell type. The total cells analyzed for each were similar between animals (see results). Raw PVH cFos counts were analyzed by counting the total cFos from four coronal sections whose rostral-caudal level was matched up between animals.

### Statistical analysis

Graphs were plotted and the statistical analyses performed using GraphPad Prism 6.0 software. One-Way ANOVA followed by Bonferroni’s post hoc test or Two-way repeated measures ANOVA was used to analyze food intake data. One-Way ANOVAs were used to compare the expression of the α4 nAChR subunit in different regions of the LH. Unpaired Student T-Tests were used to compare cFos expression. The body weights of the rats were compared using One-way ANOVA (i.p. injected rats) or Unpaired Student T-Test (intra-LH infused rats).

## Results

**Fig 1:**
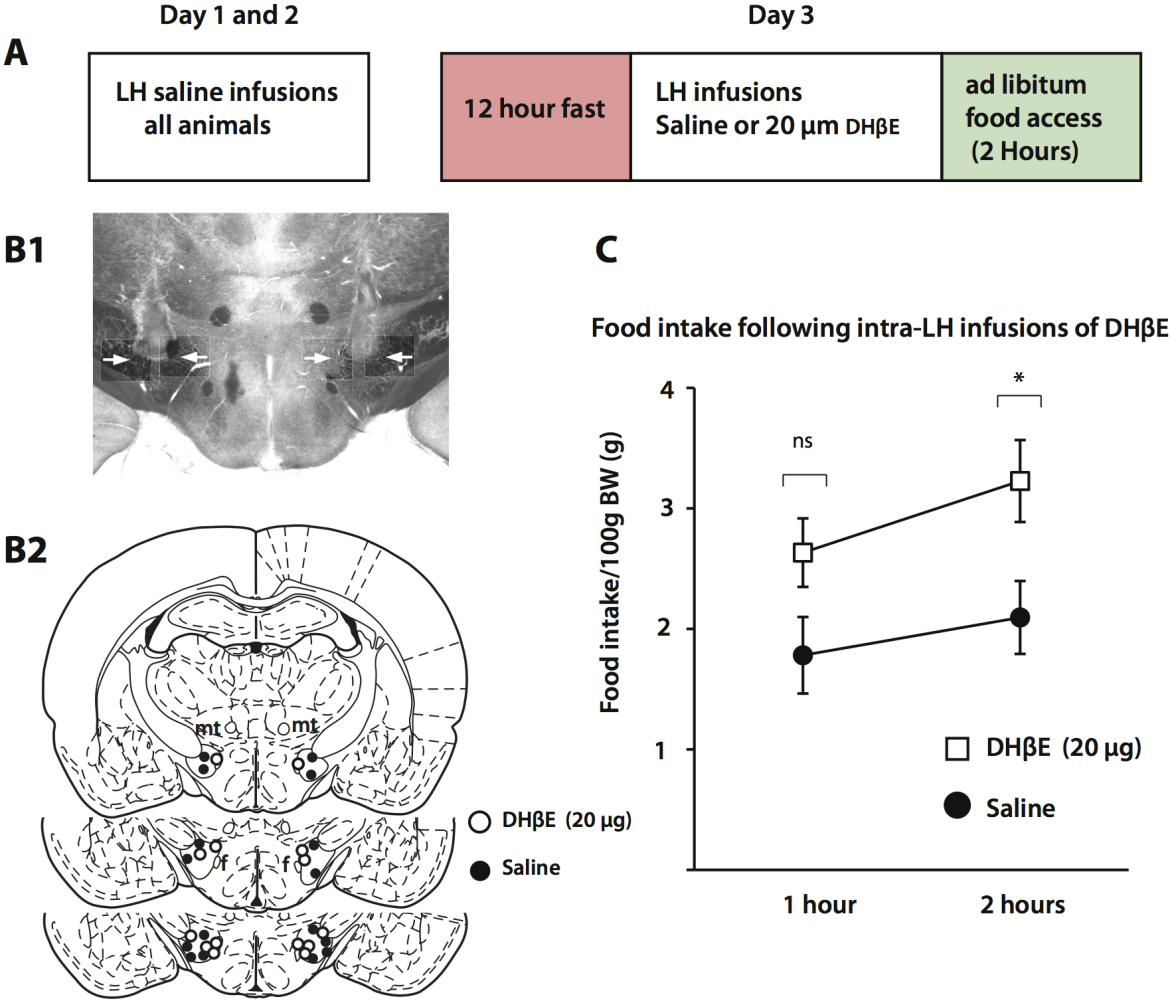
The effects of DHβE infusions into the lateral hypothalamus on food intake. A. Scheme demonstrating the experimental setup. Intra-cerebral cannulae were confined to the lateral hypothalamic region (B2) an example of which is shown in B1. Cumulative measurements of food intake showed that DHβE-infused animals consumed more food 2 hours post ad libitum food access following a 12 hour fast (C) (saline, n = 8; 20 μg DHβE, n = 7). Abbreviations: mt, mammillothalamic tract; f, fornix.

To shed light on the cholinergic influences on eating behaviour we performed bilateral intra-LH infusions of saline (n = 8) or 20 μg DHβE (n = 7), an α4 nAChR antagonist with moderate affinity, immediately following a 12 hour fast and before re-introducing ad libitum access to chow in adult Wistar rats (Fig 1). Both saline and DHβE groups were of equal weight (Saline, 371 ± 9.7 g; 20 μg DHβE, 374.6 ± 5.4 g: Unpaired Student’s T-Test, two-tailed: p = 0.812). One hour after being refed there was no difference in food intake between saline and drug-treated animals, but when food intake was measured 2 hours post ad libitum food access significant cumulative differences in food intake emerged showing that DHβE-treated animals consumed more chow than control animals (Fig 1C) (Two-way repeated measures ANOVA, dose effect, F(1,13) = 5.21, p = 0.039; time effect, F(1,13) = 28.37, p < 0.001). These results indicate that α4β2 nAChRs in the LH are physiologically engaged during normal feeding behavior to regulate food intake.

**Fig 2:**
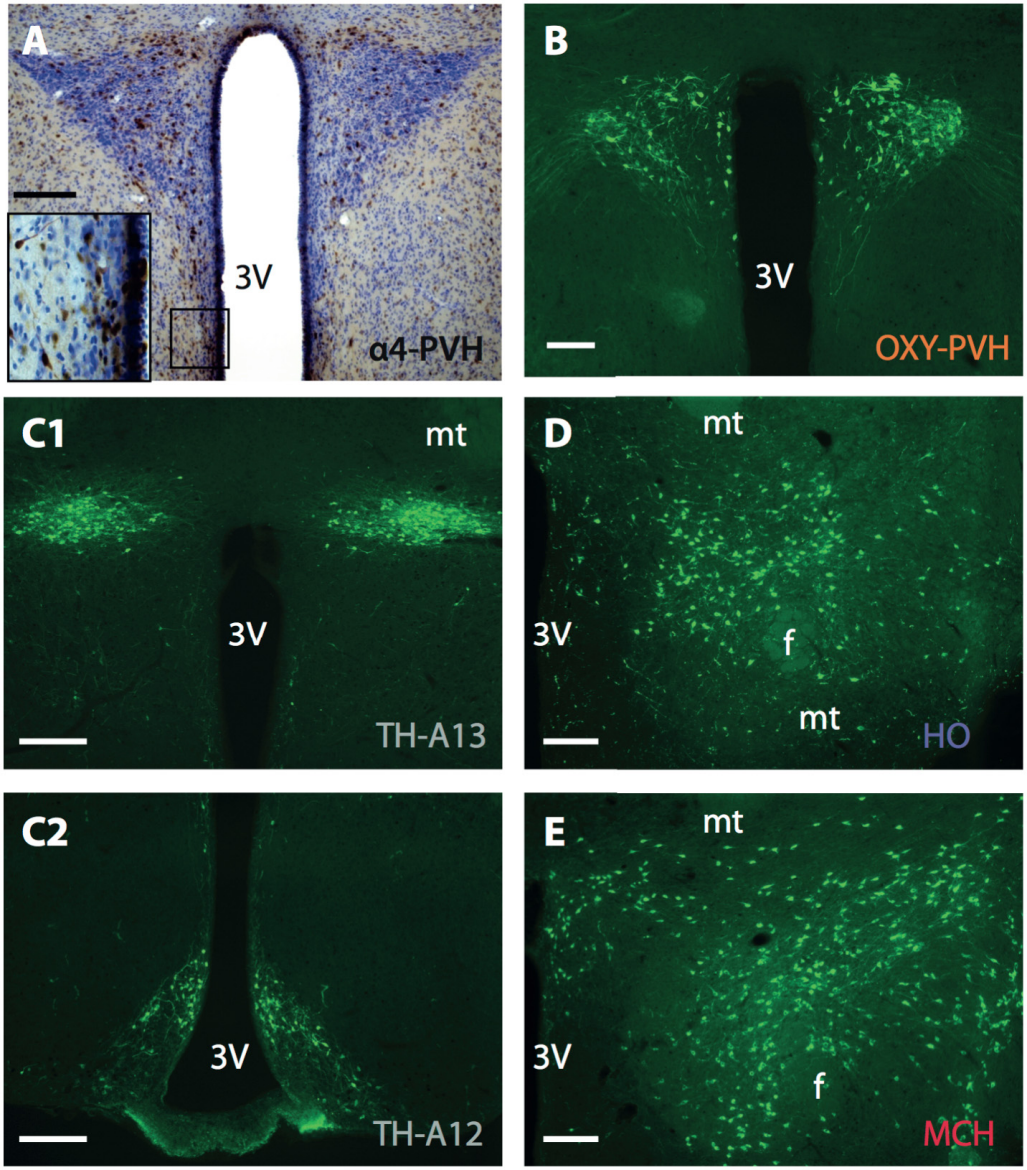
Example immunolabeling for α4 (A), oxytocin in the PVH (B), tyrosine-hydroxylase in the A13 (C1) and arcuate nucleus (C2), and HO (D1) and MCH (D2) immunoreactivity throughout the hypothalamus. Abbreviations: OXY, oxytocin; PVH, paraventricular nucleus of the hypothalamus; TH, tyrosine hydroxylase; HO, Hypocretin/Orexin; MCH, Melanin Concentrating Hormone; 3V, third ventricle; mt, mammillothalamic tract; f, fornix. Scale bar: A, B 100 μm; C-E 300 μm.

**Fig 3:**
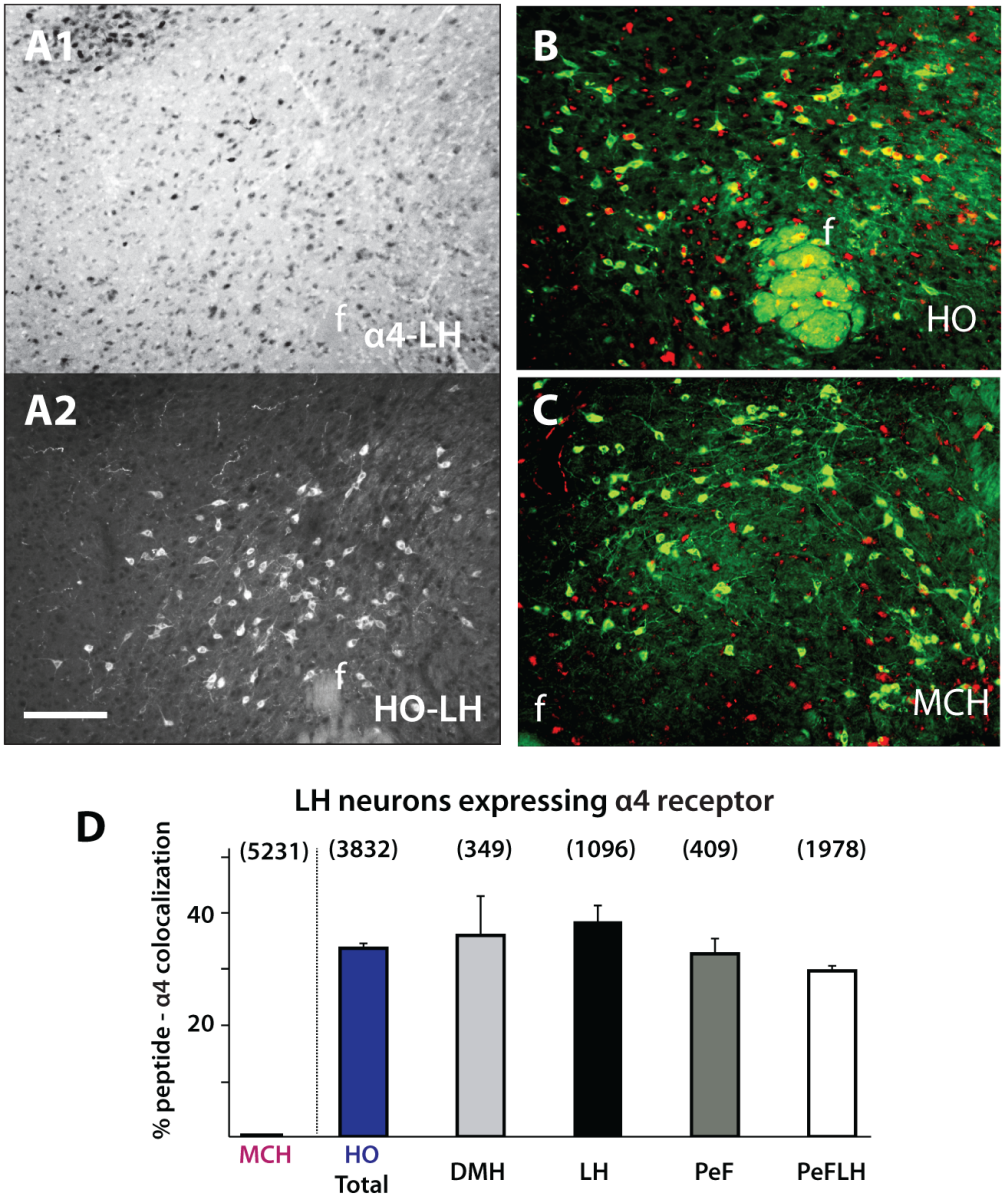
HO and MCH neurons expressing the α4 nAChR subunit. A. Example images of α4 (A1) and HO (A2) immunolabeling in the hypothalamus. 37.1% of HO neurons were colocalized with α4 (B,D) and showed no difference across various hypothalamic subnuclei (D) (α4-HO, n = 4). α4 was not seen to colocalize with MCH neurons (C,D) (α4-MCH, n = 4). Number above each column represents total cells counted. Abbreviations: HO, hypocretin/orexin; MCH, melanin concentrating hormone; f, fornix; DMH, dorsomedial hypothalamus; LH, lateral hypothalamus; PeF, perifornical region; PeFLH, perifornical region of the hypothalamus. Scale bar: 300 μm.

The α4 nAChR is moderately expressed in various hypothalamic nuclei, mostly confined to dorsal divisions including the LH, PVH, and zona incerta [38], regions that contain distinct neuronal cell types known to regulate energy balance. To shed light on the cholinergic regulation of such circuits in controlling appetite we next analyzed the expression of the α4 nAChR subunit on neurochemically defined hypothalamic circuits, specifically on HO, MCH, oxytocin, and TH containing neurons (Fig 2). Consistent with previous research, α4 immunoreactivity was seen throughout the dorsal hypothalamus (Figs 2A and 3) [38]. Interestingly, nicotine-related increases in HO activity have been previously documented but it was unclear whether or not these effects were mediated directly through HO-expressing α4β2 nAChRs. Our analysis demonstrated that 37.1% of HO neurons express the α4 subunit (n = 4), indicating that a large proportion of HO neuronal activity can be directly regulated by ACh acting via α4β2 nAChRs (Fig 3B and 3D). These percentages were consistent across different hypothalamic regions where HO neurons reside, including the dorsomedial hypothalamus (DMH), LH, perifornical region (PeF) and perifornical area of the lateral hypothalamus (PeFLH) (Fig 3B and 3D) and no significant difference between these regions was observed (One-way ANOVA, F (2, 6) = 3.180; p = 0.57). In contrast, there was no MCH-α4 nAChR co-expression (n = 4), indicating that MCH neurons are not under the direct control of the α4β2 nAChR (Fig 3C and 3D).

**Fig 4:**
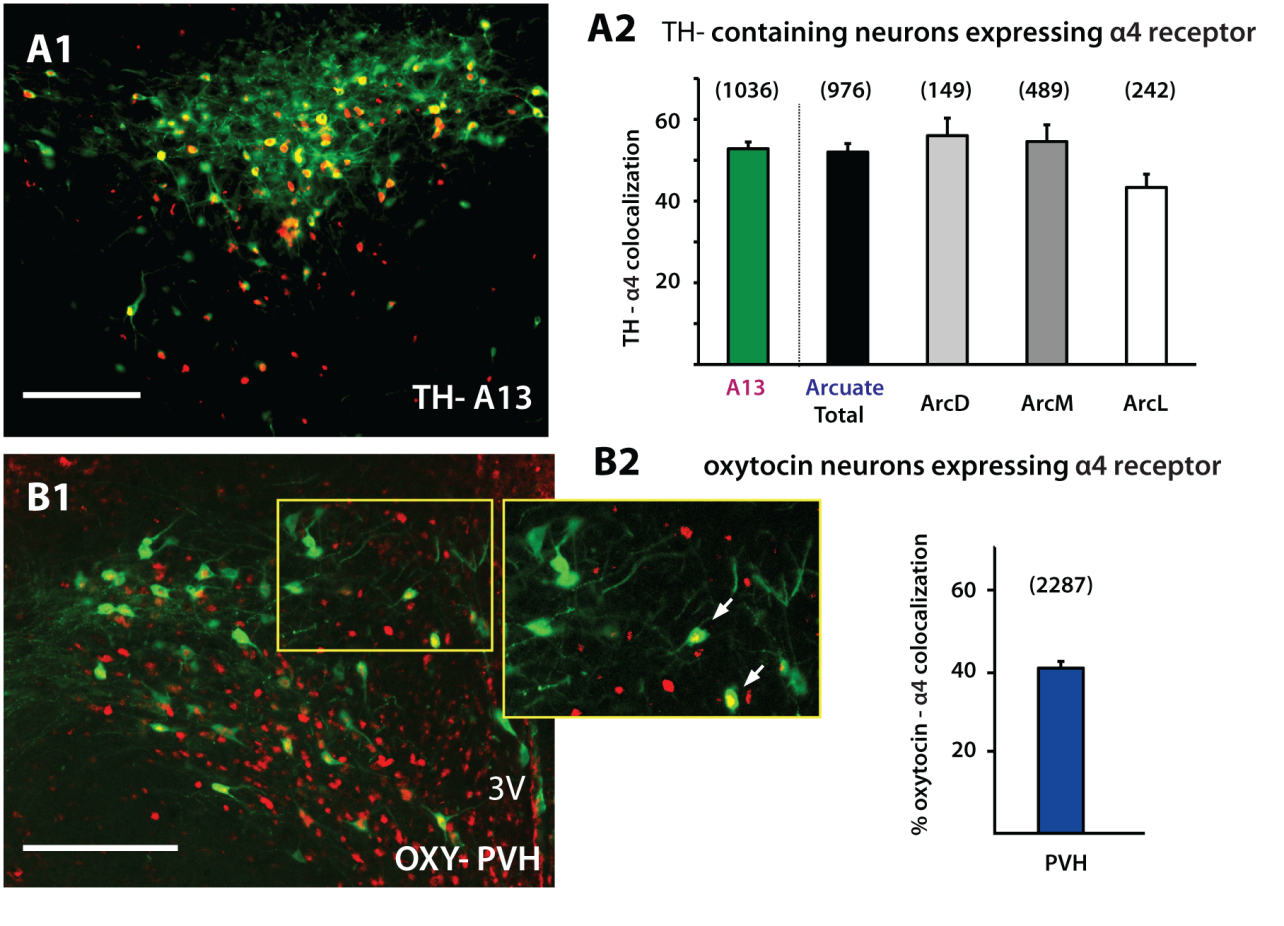
Hypothalamic TH and oxytocin neurons expressing the α4 nicotinic subunit. A. 50.1% of TH-containing neurons in the A13 were colocalized with α4 (A1,2) while 50.7% of A12 TH-containing neurons in the arcuate nucleus showed α4 nAChR co-expression (A1,A2) (α4-TH, n = 3). There was no difference in colocalization across the different arcuate subdivisions analyzed (A2). B. Within the PVH, 41.3% of oxytocin neurons were also immunolabeled for α4 (α4-oxy, n = 4). Abbreviations: TH, tyrosine hydroxylase; Oxy, oxytocin; PVH, paraventricular nucleus of the hypothalamus; Arc, Arcuate nucleus; 3V, third ventricle. Scale bar: 200 μm.

Our observations revealed strong α4 immunoreactivity in the hypothalamus including the PVH (Fig 4B1). In terms of the regulation of appetite, one candidate cell type potentially under cholinergic regulation is that of neurons expressing oxytocin [9]. Increases in both PVH oxytocin activity and release in the hypothalamus have been reported to cause a decrease in food intake [9–10, 39]. Our analyses confirmed that ACh can act on oxytocin neurons located in the PVH since 41.3% of these neurons were also immunopositive for the α4 subunit (n = 4) (Fig 4B). Dopamine neurons in the mesolimbic system have been shown to express and be under the influence of α4β2 nAChRs [40–41]. In light of this we next analyzed hypothalamic dopamine populations located in A13 of the zona incerta (n = 3) and A12 of the arcuate nucleus (n = 3). Both populations showed similar levels of α4 expression (50.1% and 50.7%), TH-expressing neurons in A12 showed no difference in colocalization across the different arcuate subdivisions analyzed (Fig 4A) (One-Way ANOVA, F (2, 6) = 3.180, p = 0.11). Overall, in all identified neurons analyzed in this study TH-containing neurons showed the highest degree of colocalization with the nicotinic α4 subunit.

**Fig 5:**
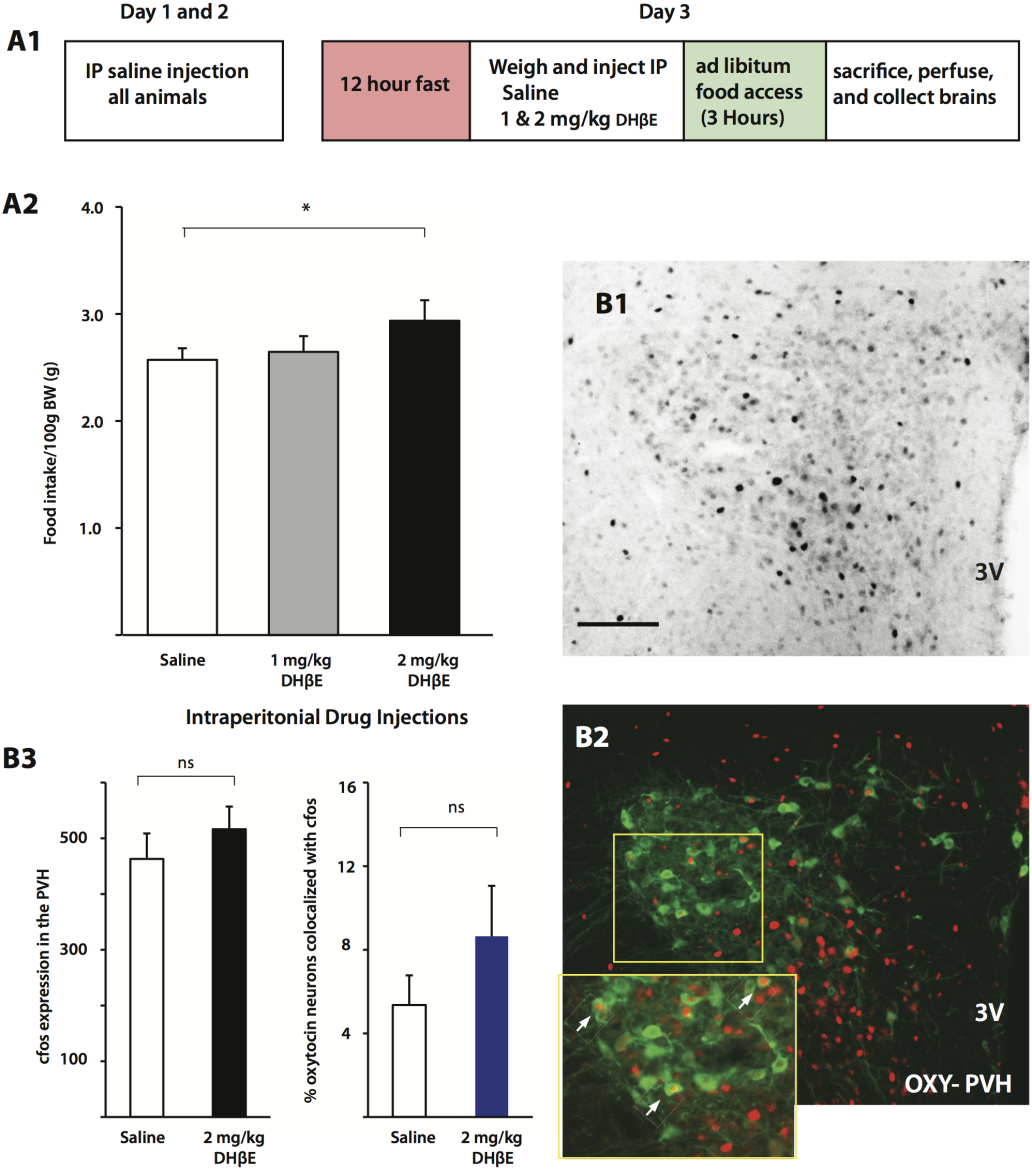
The effects of systemic DHβE injection on food intake and PVH activity. A1. Schematic demonstrating the experimental setup. A2. Animals injected with 2 mg/kg DHβE i.p. consumed more food than saline injected animals (saline, n = 7; 1 mg/kg, n = 7; 2 mg/kg, n = 6). Total cFos (B1) (Saline and DHβE, n = 5) and percent of PVH oxytocin-expressing neurons (B2) (Saline and DHβE, n = 4) were not significantly different between DHβE and Control conditions (B3). Abbreviations: PVH, paraventricular nucleus of the hypothalamus; OXY, oxytocin 3V, third ventricle. Scale bar: 100 μm.

To shed light on the hypothalamic appetite circuits potentially regulated by ACh we repeated our feeding experiment but instead of the LH infusion of DHβE we injected animals i.p. with DHβE (see Fig 5A1). The animals’ weights from the three groups (Saline, 1 mg/kg, and 2 mg/kg) were not significantly different (Saline, 356.5 ± 8.1 g; 1 mg/kg, 361.6 ± 6.4 g; 2 mg/kg, 354.0 ± 6.9 g: One-way ANOVA: F_2,17_ = 0.29, p = 0.75). Similar to the case of intra-LH infusions of DHβE, i.p. injections of 2 mg/kg DHβE (n = 6) resulted in animals consuming more food compared to saline injected animals (n = 7) 2 hours after fasted animals were given free access to food, whereas 1 mg/kg DHβE (n = 7) had no effect (Fig 5A2) (Two-way ANOVA: F_2,17_ = 4.63, Bonferonni post-test, 2 mg/kg vs. saline, p = 0.028). In animals receiving injections of saline and 2 mg/kg of DHβE, differences in activity levels in neurochemically identified neurons were quantified by immunolabeling for cFos, an immediate early gene product that is often used as a marker for cell activation. Cellular expression of cFos is confined to the nucleus, making its expression in immunolabeled neurons clearly visible (Fig 5B2, inset) and typically reaches maximum value between 90 to 180 minutes after cellular activation [42]. To capture activation patterns based on differences in satiety levels between the groups we perfused animals 3 hours after re-introducing food. Analysis of raw PVH cFos counts in saline and DHβE conditions (Saline, n = 5, 463 ± 46 cells; DHβE, n = 5, 516 ± 41 cells: Unpaired Student’s T-Test, two-tailed: p = 0.42) did not show any significant differences (Fig 5B1 and 5B3) nor did analysis of oxytocin-expressing PVH neurons (Fig 5B2 and 5B3) (n = 4; Saline, 5.36 ± 1.42%, DHβE, 8.65 ± 2.41%: Unpaired Student’s T-Test, two-tailed: p = 0.28) (Fig 5B).

**Fig 6:**
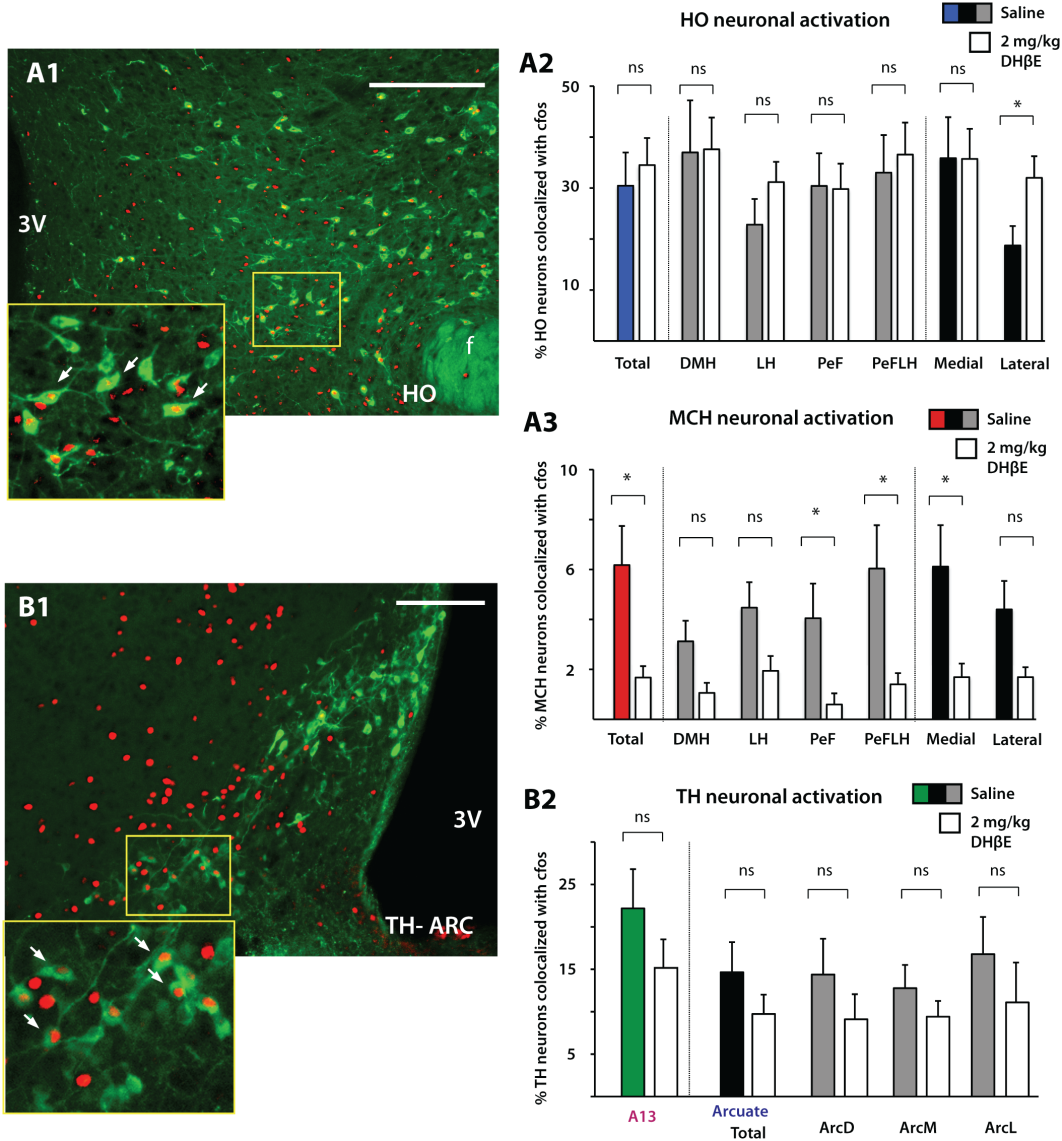
The effects of systemic DHβE injections on HO, MCH, A13-TH and A12-TH activity. In animals that showed greater food intake compared to controls, DHβE injections at 2 mg/kg increased cFos expression in laterally located HO neurons (A1-2) (Saline and DHβE, n = 6) and decreased cFos expression in total MCH neurons (A3) (Saline, n = 4; DHβE, n = 5). There was no difference between 2 mg/kg DHβE and Saline groups in TH A13 (Saline and DHβE, n = 6) and A12 cFos expression (Saline and DHβE, n = 4) (B1-2). Abbreviations: HO, hypocretin/orexin; MCH, melanin concentrating hormone; TH, tyrosine hydroxylase; f, fornix; DMH, dorsomedial hypothalamus; LH, lateral hypothalamus; PeF, perifornical region; PeFLH, perifornical region of the hypothalamus; Arc, arcuate nucleus. Scale bar: A, 300 μm; B, 100 μm.

As our intracranial injections were mostly confined to the LH (Fig 1A) we next shifted our attention to distinct populations centered on the LH. Nicotine administration has been shown to increase the expression of cFos in HO neurons, suggesting that HO activity can be enhanced by endogenous ACh acting via nAChRs [24]. In their study nicotine-mediated increases in HO activity were blocked by the addition of the nAChR antagonists mecamylamine and DHβE, suggesting that the nicotine-mediated increase in HO activity was likely via a heteromeric αβ nAChR pathway. Our results demonstrating that HO neurons express the nicotinic α4 subunit are consistent with the idea that nicotine can directly excite HO neurons by acting on a functional α4β2 cationic channel. To investigate the impact of DHβE in the context of food intake we analyzed the activity of HO neurons in the control (n = 6; 30.46 ± 6.51% of HO cells expressing cFos) and 2mg/kg DHβE (n = 6; 34.50 ± 5.30% HO cells expressing cFos) groups. No significant differences between the saline-treated and 2mg/kg DHβE-treated groups were found in the % colocalization of cFos immunoreactivity in HO neurons, nor across the various hypothalamic subregions (Fig 6A2) (Unpaired Student’s T-Test, two-tailed: Total, p = 0.65; DMH, p = 0.95; LH, p = 0.22; PeF, p = 0.94; PeFLH, p = 0.72). However, when we divided the HO population into a medial and lateral division, surprisingly more HO neurons in the 2mg/kg DHβE group expressed cFos, indicating that under DHβE conditions resulting in greater food intake, laterally located HO were found to be more active (Fig 6A2, far right) (Medial; Saline 35.86 ± 8.04%, DHβE 35.72 ± 5.9%: Lateral; Saline 18.73 ± 3.82%, DHβE 32.01 ± 4.24: Unpaired Student’s T-Test, two-tailed: Medial, p = 0.99; Lateral, p = 0.04). Our results demonstrating greater HO activation when α4β2 nAChRs are antagonized are inconsistent with results by Pasumarthi et al. [24] since in their study, nicotine-related increases in HO cFos expression are lower with i.p. injections of DHβE. This difference could result from the fact that rather than activating nAChRs with exogenous nicotine, our study was designed to disrupt normal cholinergic transmission acting via nAChRs, which as a consequence led to significant behavioral changes in food intake.

Our α4 nAChR immunolabeling results show that MCH neurons do not express the α4 subunit (Fig 3C), indicating that MCH neurons are not under the direct influence of α4β2 nAChRs. Nevertheless we next analyzed cFos expression patterns in MCH neurons under both saline (n = 4) and 2mg/kg DHβE (n = 5) conditions. HO and MCH neurons show complementary functions in relation to homeostatic processes such as energy homeostasis and we thus predicted that an increase in HO activity would be complemented by a decrease in MCH activity. Indeed, our results demonstrate an overall decrease in total MCH activity in the 2mg/kg DHβE-group compared to saline-injected animals (Fig 6A3) (Saline 5.24 ± 1.22%, DHβE 1.67 ± 0.46%: Student’s T-Test, two-tailed, p = 0.03) (Fig 6A3). Significant differences were confined to the PeF, PeFLH and medial regions (Student’s T-Test, two-tailed: PeF, p = 0.04; PeFLH, p = 0.04; Medial, p = 0.03) while in other divisions there was no significant difference between saline and 2 mg/kg DHβE injections (Student’s T-Test, two-tailed: LH, p = 0.07; DMH, p = 0.07; Lateral, p = 0.07) (Fig 6A3). These results are consistent both in vivo and in vitro studies showing that when HO activity is elevated, MCH neuronal activity is reduced [43–44]. Analysis of cFos in dopaminergic A13 (for both groups n = 6; Saline 22.17 ± 4.62%, DHβE 15.16 ± 3.36%) and A12 (for both groups n = 4; Saline 14.65 ± 3.55, DHβE 9.74 ± 2.28%) cell groups showed no difference between saline and 2 mg/kg DHβE injection groups (Fig 6B) (Student’s T-Test, two-tailed: A13 total, p = 0.26; A12 total, p = 0.288; ArcD, p = 0.36; ArcM, p = 0.34; ArcL, p = 0.41) although in both TH groups, cFos immunoreactivity in the 2 mg/kg DHβE group was overall lower than in the control.

## Discussion

Here we show that a variety of neurochemically distinct hypothalamic circuits express the α4 nicotinic subunit and antagonizing the α4β2 nAChR in the lateral hypothalamus of fasted animals led to an increase in food intake compared to vehicle infused animals. Systemic DHβE administration led to similar results and were accompanied by an increase in HO neuronal activity in the lateral division of the hypothalamus and an overall decrease in total MCH activity as measured by immunolabeling for cFos. No change in activity was seen in PVH oxytocin neurons or in A13 and A12 TH-containing neurons.

Our results revealing changes in HO and MCH activity in i.p. DHβE-injected animals is consistent with our initial behavioral results as both MCH and HO populations predominantly reside in the LH thus supporting previous work showing that cholinergic transmission in LH networks can affect metabolic functions [28]. Despite being strongly immunoreactive for the α4 subunit of the excitatory α4β2 nAChR and contrary to what one would expect to occur when antagonizing a known excitatory cationic channel, systemic DHβE administration led to greater activity in laterally located HO neurons, a model supported by work done by Pasurmarthi et al. [24] showing that acute nicotine injections dose-dependently increase cFos expression in OH neurons. In the context of naturally occuring feeding behavior, our results suggest that HO neuronal activity may not be influenced directly by the cholinergic activation of α4β2 nAChRs but rather is indirectly affected by ACh impacting both HO and MCH neurons through local hypothalamic cell populations. Consistent with this, our α4 immunolabeling data showed that in addition to HO neurons the α4 nAChR subunit was expressed throughout the LH (Fig 2, data not quantified) and despite MCH neurons being immunonegative for the α4 nAChR subunit there was a sharp decrease in MCH activity in the 2 mg/kg DHβE group. Together this data supports the proposition that DHβE-mediated increases and decreases in HO and MCH activity, respectively, may result from a decrease in local inhibitory and excitatory drive onto HO and MCH neurons stemming from other hypothalamic circuits. This can potentially occur by ACh acting locally in the LH to neuromodulate LH circuits by influencing dopamine and serotonin release [19, 45] but also by directly influencing local excitatory and inhibitory networks. Previous in vitro studies have shown that bath-application of nicotine can act presynaptically to enhance glutamatergic [46] and GABAergic [23] transmission in the hypothalamus, the latter of the two cases by acting on αβ nAChRs. Cholinergic inputs to the LH may therefore be regulating food intake via distinct hypothalamic circuits that may ultimately impact HO and MCH neuronal activity. Two recent studies have shown that photoactivating vGlut2- [47] or vGAT [48], presumably glutamatergic and gabaergic, expressing neurons in the LH can decrease and increase food intake, respectively. Whether or not these circuits are also influenced by αβ nAChRs remains to be seen.

Converging melanocortin circuits onto chemically distinct PVH neurons are critical for regulating appetite [39, 49–50]. Here we show significant α4 immunoreactivity in PVH (Fig 2A) neurons, suggesting that these cells are strongly influenced by ACh acting via αβ nAChRs. Our activity analysis however revealed no differences in PVH cFos expression between the groups or in hypothalamic TH-containing neurons despite these neurons showing the greatest α4 immunoreactivity. This perhaps may be due to the inherent sampling restrictions and low temporal resolution of tracking the timecourse of cFos expression. Alternatively, considering that direct LH infusions of DHβE affected food intake, DHβE-mediated increases in consummatory behavior may arise independently of PVH or hypothalamic TH processing.

Disruptions in the HO system serve to decrease appetite and profoundly reduce physical activity, indicating that the obese phenotype previously documented in animals with such disruptions results mostly from a decrease in energy expenditure. In addition to increasing food intake, intracerebral infusions of HO are known to increase spontaneous activity and exercise on a running wheel [51], together establishing that the HO circuits may be important in initiating active measures to seek out food for maintaining energy homeostasis. Indeed, HO cells are stimulated during hypoglycemia [52] and are inhibited when glucose levels are high [53]. Moreover, HO neurons have been shown to increase their activity when food-restricted animals are entrained to anticipate food delivery, thereby increasing locomotor activity associated with food seeking. A recent study has shown that more laterally located HO neurons in the PeF and LH are preferentially engaged during consummatory behavior while HO neurons located medially in the DMH are mostly active during the food anticipatory phase prior to feeding [54]. These results are consistent with a model proposed by Harris and Aston-Jones [55] suggesting a functional dichotomy between medially located HO neurons in the DMH regulating arousal with laterally located HO neurons being more engaged during consummatory behavior. Our results showing increases in laterally located HO populations are consistent with this scheme since DHβE-injected animals consumed more than control animals, a behavioral effect that was recapitulated when DHβE was directly infused into lateral divisions of the hypothalamus.

The most profound change in cellular activity was seen in MCH neurons, with 2 mg/kg DHβE-injected animals showing decreases in total MCH activity. HO and MCH neurons are known to have opposing response profiles [43] as well as behavioral actions promoting food seeking and energy conservation, respectively [56]. Actions of both transmitters are known to increase food intake although the consummatory drive is thought to stem from separate metabolic requirements. Much evidence supports the idea that MCH neurons reinforce consumption of palatable food for the purpose of storing and conserving energy while the HO system is more important in initiating food seeking behavior and consumption. Our cFos analysis capturing an increase in a subpopulation of HO neurons and decrease in MCH activity may reflect the initial phase of consummatory behavior in DHβE-treated animals, but whether or not these activity patterns would change over time to reflect the prolonged meal duration and thus conservation of energy would require further studies with different cFos sampling times. Nonetheless, the results of the current study indicate that a number of different hypothalamic circuits are under the influence of α4-containing nAChRs and that endogenous cholinergic inputs to the hypothalamus can regulate food intake by acting on α4β2 nAChRs. These effects on satiety signals may result from the activation of local LH networks that differentially influence the activity of neurochemically distinct hypothalamic populations known to be important in maintaining energy homeostasis.

## Supporting Information

S1 Checklist PLOS one ARRIVE checklist. (TIFF)
