# Supplementary figures and images for: Nicotinic α4 Receptor-Mediated Cholinergic Influences on Food Intake and Activity Patterns in Hypothalamic Circuits
Source: PLoS One. 2015 Aug 6;10(8):e0133327. doi: 10.1371/journal.pone.0133327 (PMC4527587; doi:10.1371/journal.pone.0133327)

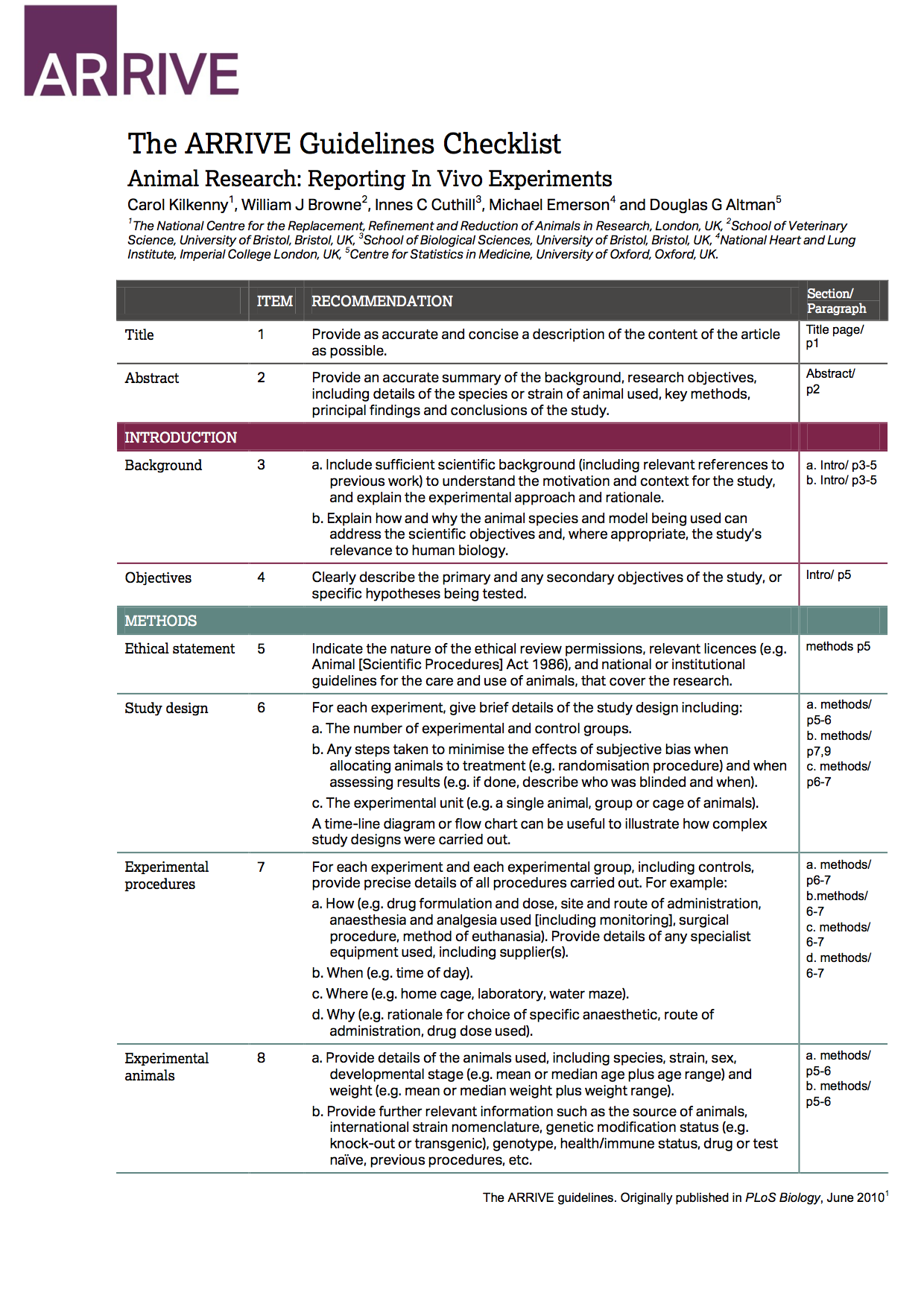

Supplement: S1 Checklist — (TIFF) [file pone.0133327.s001.tiff]
